# Spontaneous peripheral artery rupture in patients with neurofibromatosis type 1

**DOI:** 10.1016/j.jvscit.2025.101873

**Published:** 2025-06-06

**Authors:** Luyang Che, Yangyang Ge, Yongle Xu, Xiaoping Liu, Shaoliang Luan, Wei Guo

**Affiliations:** a Department of Vascular and Endovascular Surgery, Chinese PLA General Hospital, Beijing, China; b Department of Vascular and Endovascular Surgery, Hainan Hospital of Chinese PLA General Hospital, Sanya City, Hainan Province, China

**Keywords:** Neurofibromatosis, NF1, Arterial rupture, Endovascular arterial repair

## Abstract

Artery rupture is a very rare but severe complication of neurofibromatosis type 1 (NF1), an autosomal-dominant genetic disorder, and the optimal treatment is not yet clear. We present two cases who presented with different types of artery rupture related to NF1 at our center within a short period and review similar cases reported previously to discuss the suitable therapy for this complication. We concluded that an NF1 gene mutation affects the structure of the artery, makes the artery fragile, thick, and prone to rupture, and endovascular treatment should be considered as the first choice.

Neurofibromatosis type 1 (NF1), also known as von Recklinghausen's disease, is an autosomal-dominant genetic disorder, affecting 1 in 3000 individuals and accounting for 96% of all cases of NF.^1^ Symptoms of NF1 include distinctive skin changes, bone changes, and cognitive impairment.^2^ Artery rupture is a very rare complication of NF1. In the past, NF1-related arterial rupture has been published as case reports only, and there were few systematic studies and summaries of its pathogenesis, pathological process, and treatment. Recently, we treated two NF1-related artery rupture patients within 1 month, which is more than that we encountered in the past 10 years combined. The association between these patients remains unknown, and an influence of temperature change in a short period owing to weather fluctuations was suspected.^3^ We report these cases and review the literature. Both patients have given their explicit consent for publication.

## Case report

### Case 1

**Fig 1:**
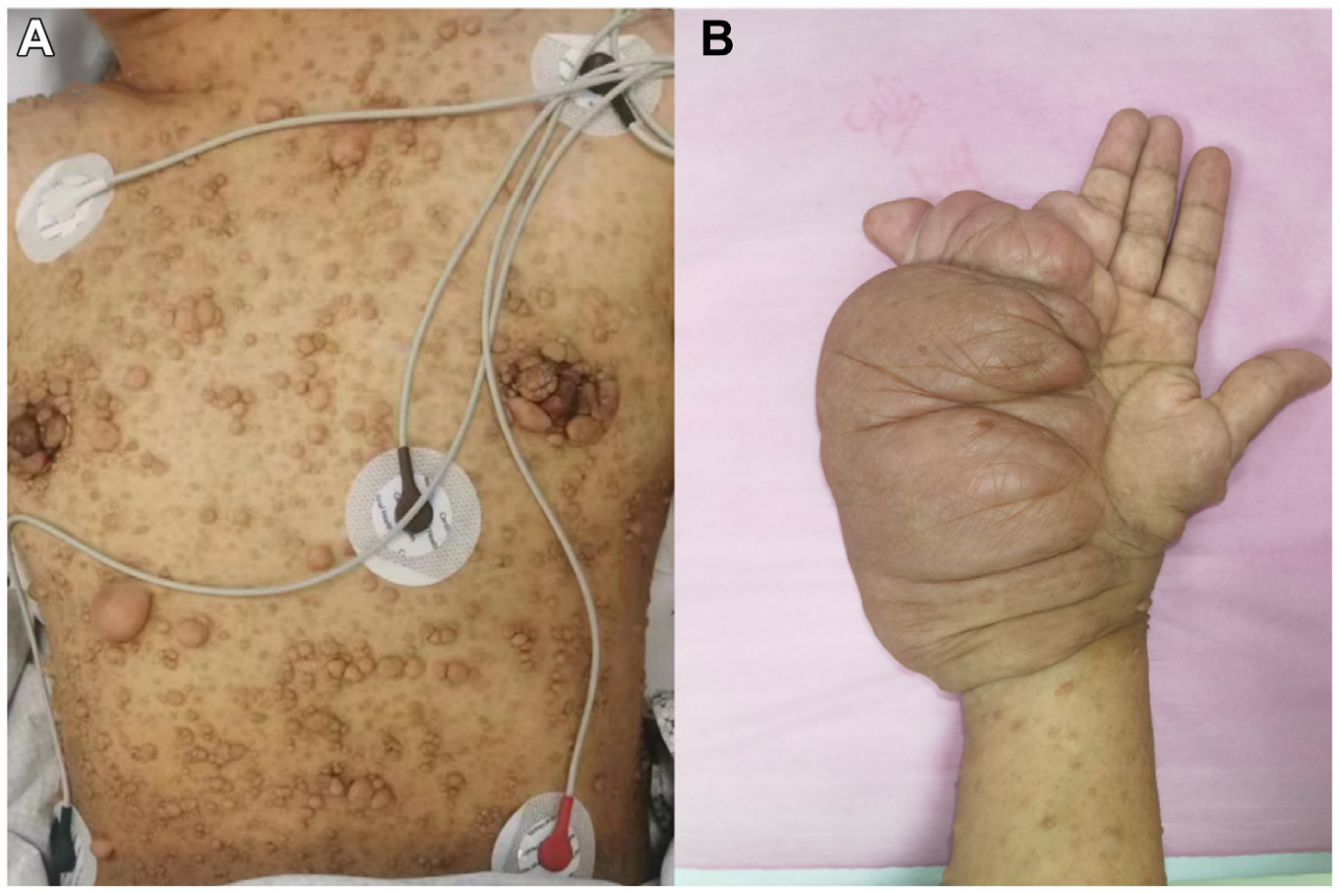
Physical examination features of patient 1. **(A)** Numerous tumor-like nodules of different sizes widely distributed on the patient's skin. **(B)** Enlargement and malformation of the patient's left palm.

**Fig 2:**
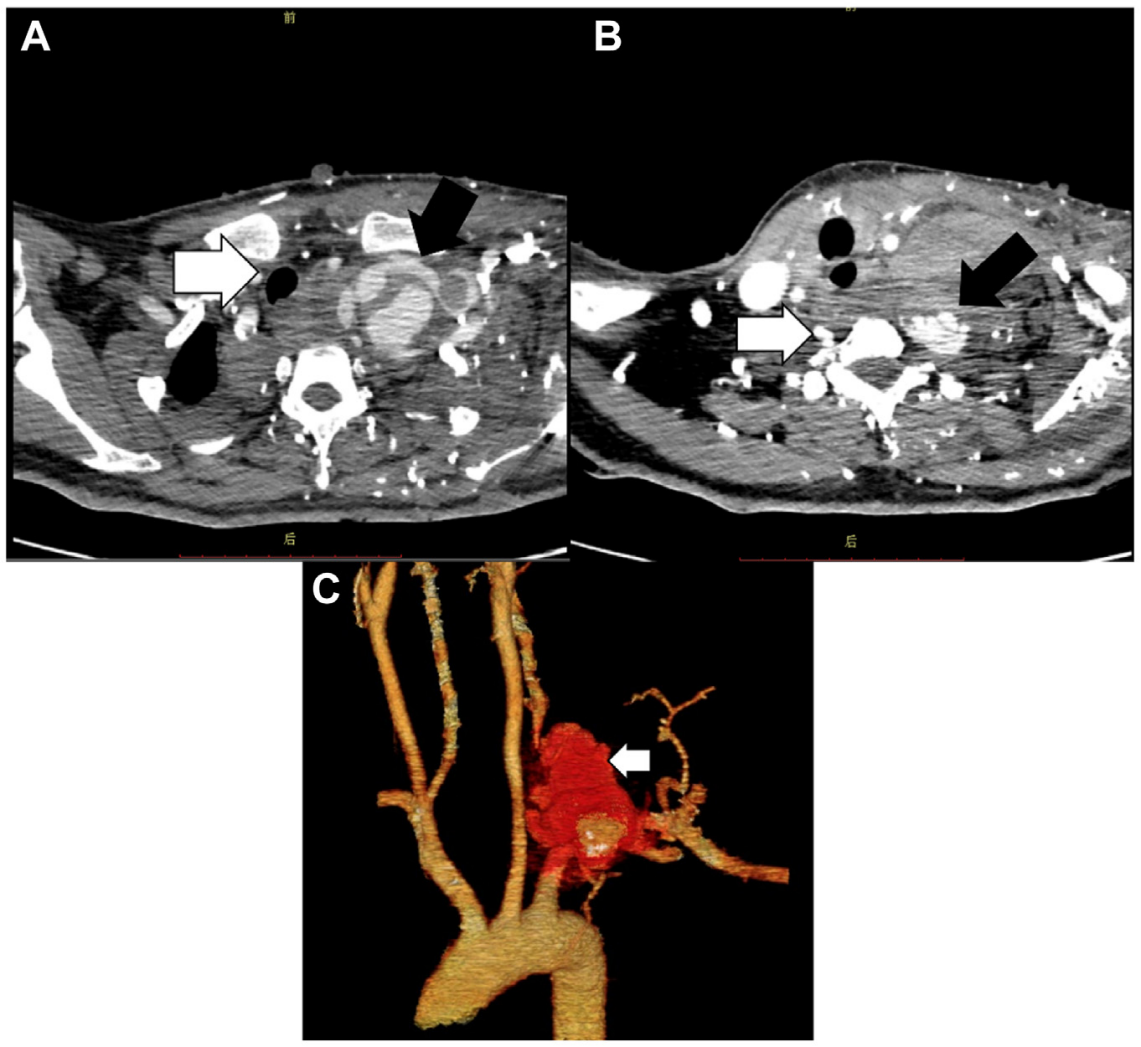
Computed tomography arteriography (CTA) images of case 1. **(A)** A large hematoma (*black arrows*) can be observed in the patient's thorax, the mediastinum is broadened, and the trachea was deviated to right (*white arrow*). **(B)** Suspicious rupture of the left vertebral artery. Another hematoma can be seen near the left vertebral artery (*black arrow*), whereas the right vertebral artery is normal (*white arrow*). **(C)** Three-dimensional reconstructed image showing a massive hematoma (*white arrow*) surrounding the left vertebral artery and the left subclavian artery (LSA).

A 37-year-old woman presented with a swollen neck and a mass formation after physical labor. She was diagnosed with NF1 20 years ago and received no treatment for personal reasons. Her father and grandfather were also diagnosed with neurofibromatosis; however, her mother was healthy. The patient was conscious and had numerous tumor-like skin nodules of different sizes widely distributed on her face, trunk, and limbs (Fig 1, *A*). The patient's left wrist and palm were malformed, thus limiting her hand function (Fig 1, *B*). Computed tomography arteriography (CTA) revealed a massive hematoma extending from her left neck to her left mediastinum, suggesting a possible bleeding (Fig 2). Her laboratory values were all within normal limits, and she denied having a history of unexplained fever, surgery, or trauma.

**Fig 3:**
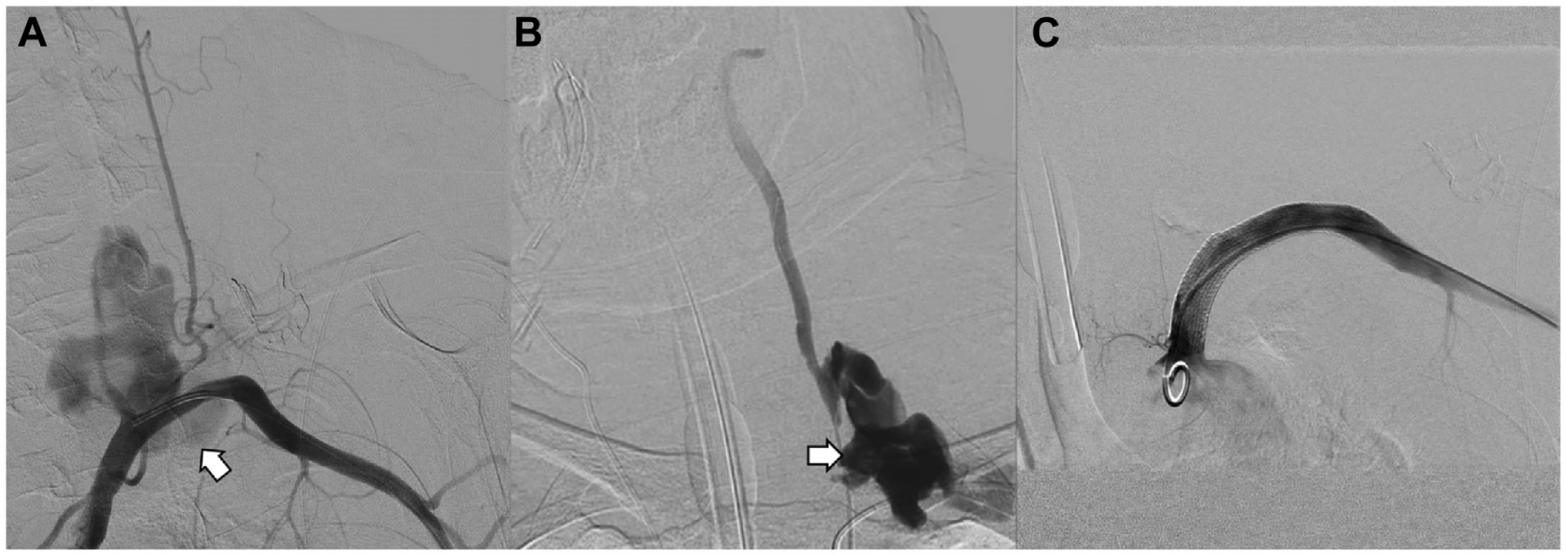
Angiography images of case 1. **(A)** Rupture site (*arrow*) of the left subclavian artery (LSA). **(B)** Rupture site (*arrow*) of the left vertebral artery with reverse blood flow. **(C)** Angiography image after stent implantation and coil embolism, showing no contrast extravasation.

**Fig 4:**
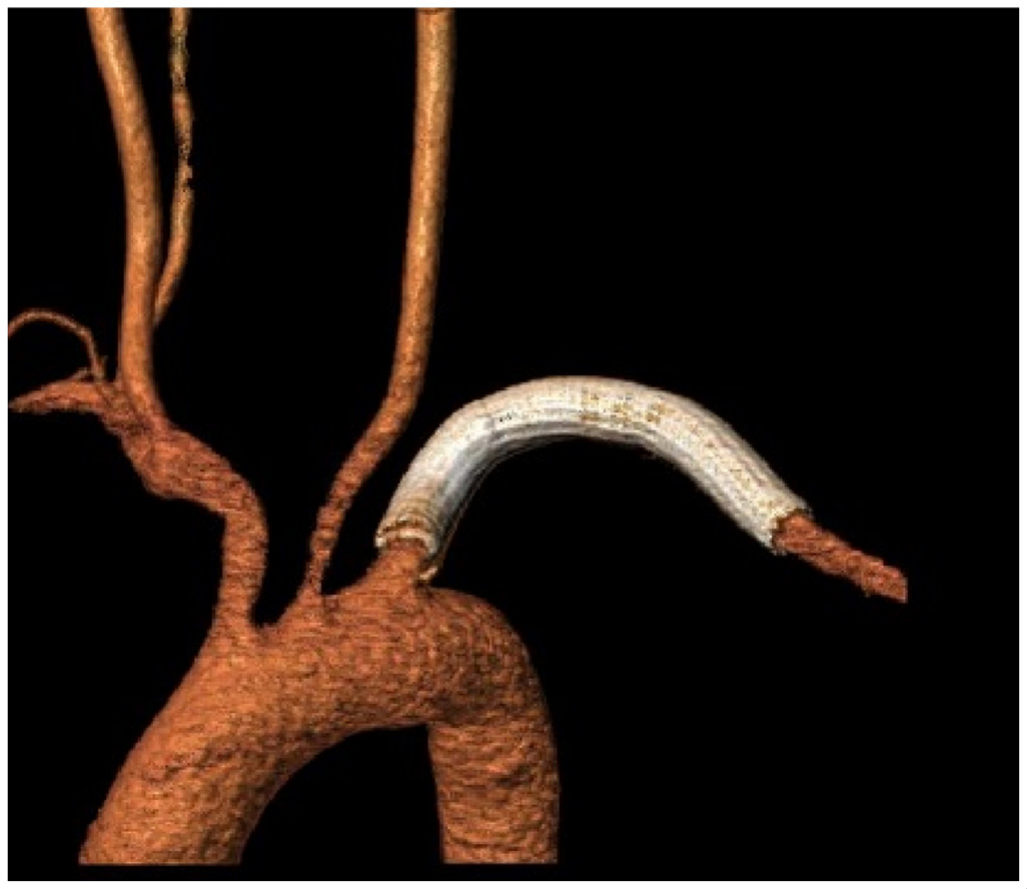
Three-dimensional reconstruction of computed tomography arteriography (CTA) image for case 1 at 1 month after the endovascular procedure, revealed normal left subclavian artery (LSA) flow.

An emergency endovascular procedure was performed under local anesthesia. Two artery rupture sites were found on the left subclavian artery (LSA) and the left vertebral artery separately (Fig 3, *A* and *B*). Reverse flow was noticed on the left vertebral artery reflecting steal physiology. Two interlocks (Boston Scientific Co., Natick, MA) were embolized in the distal left vertebral artery to block the reverse blood flow, and a 10/100 mm Viabahn stent (W. L. Gore & Associates, Newark, DE) was placed in the LSA to repair the rupture. Arteriography after stent implantation revealed no contrast media extravasation at either rupture sites (Fig 3, *C*). Dual antiplatelet therapy (aspirin + clopidogrel) was administered for 6 months, followed by long-term single-agent antiplatelet therapy. CTA examination 1 month after surgery revealed normal LSA flow and no signs of contrast extravasation (Fig 4). The patient reported no discomfort at the 6- and 12-month telephone follow-ups.

### Case 2

**Fig 5:**
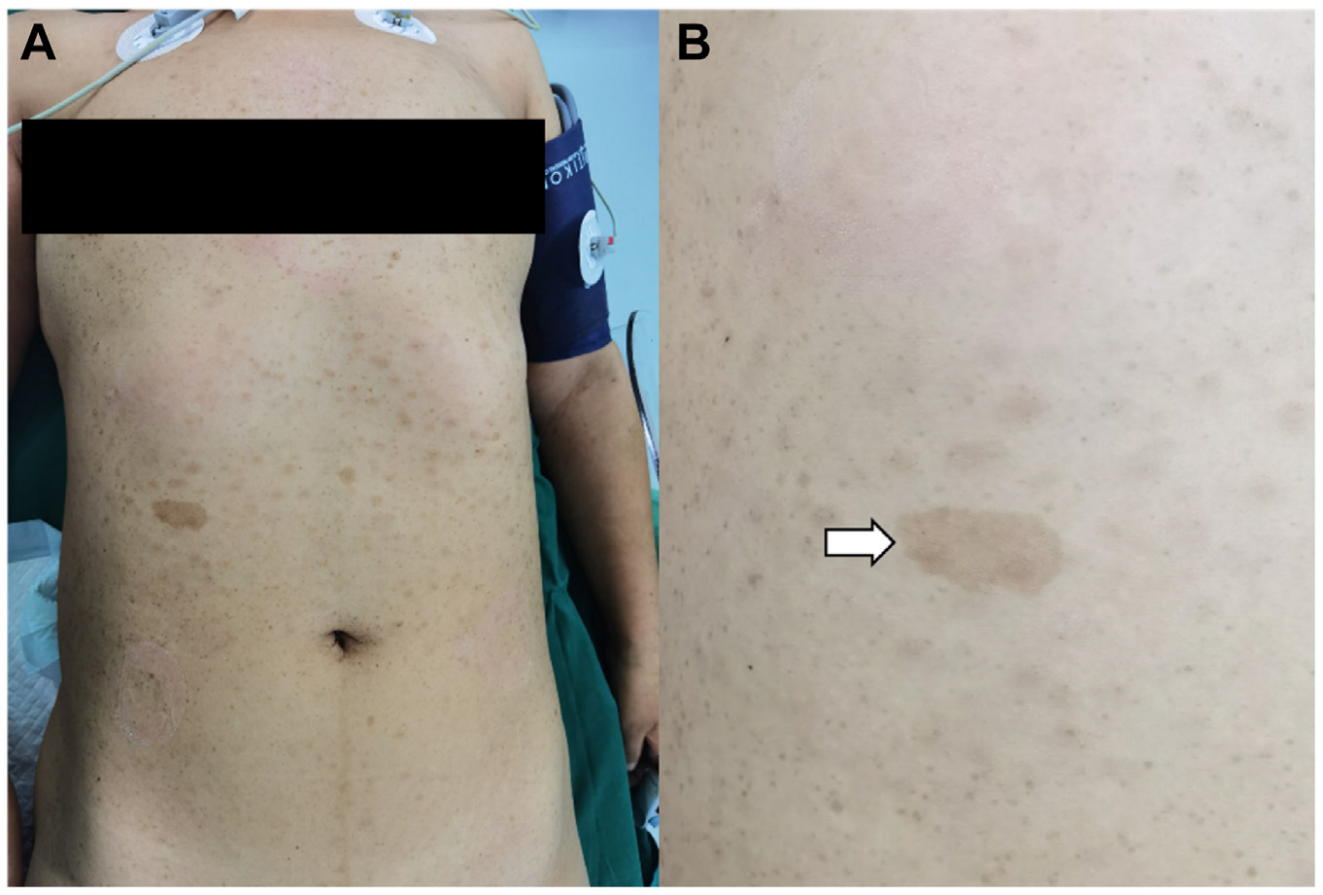
Skin appearance features of case 2. **(A)** Multiple café-au-lait spots can be seen on the patient's trunk. **(B)** Enlarged photo to show the detail of the café-au-lait spot (*white arrow*).

**Fig 6:**
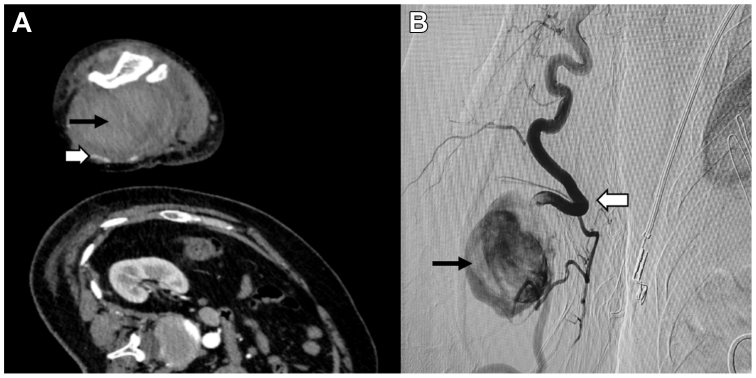
Computed tomography arteriography (CTA) and digital subtraction angiography (DSA) image of case 2. **(A)** A large hematoma (*black arrows*) can be seen on the patient's right upper arm. The right brachial artery was tortuous and was compressed by the hematoma (*white arrow*). **(B)** DSA image showing a tortuous brachial artery (*white arrow*) with contrast extravasation, indicating arterial rupture and pseudoaneurysm formation (*black arrow*).

A 39-year-old woman was diagnosed with a brachial artery rupture at a local hospital. Her arm continued swelling after conservative treatment for 20 days. She was conscious when she arrived at our center, and physical examination revealed multiple café-au-lait spots (Fig 5) and obvious swelling of her right upper arm with skin tension and ecchymosis. The patient was diagnosed with NF1 at 4 years old and no treatment was undertaken because she had been asymptomatic. Both her mother and grandmother were diagnosed with NF. CTA examination revealed a large hematoma in her right upper arm and an evident tear in the distal brachial artery (Fig 6, *A*). Her blood test revealed a hemoglobin level of 108 g/dL; leukocyte and platelet counts were within the normal range. The patient had a normal body temperature and denied having a history of unexplainable fever, surgery, or trauma.

Arteriography under general anesthesia revealed a rupture of a tortuous right brachial artery with a pseudoaneurysm (Fig 6, *B*), which was consistent with the CTA. Endovascular repair was attempted, but the tortuous artery could not be navigated. We decided to make an incisional repair. After the proximal branchial artery was occlusion with a 7/40-mm balloon (Biotronik, Lake Oswego, OR), a longitudinal incision was made on the right upper arm and multiple thrombi were removed. Attempts at an end-to-end artery anastomosis failed because of the fragility and thinness of the arterial wall. Ultimately, we endovascularly embolized the proximal brachial artery with three interlocks (Boston Scientific Co., Natick, MA) and sutured the distal end of the brachial artery with the surrounding tissues. Approximately 1500 mL of blood was lost perioperatively, and approximately 900 mL of red blood cells, 800 mL of plasma, and 2 U of prothrombin complex were transfused. The patient was discharged 7 days after surgery. Ultrasound examination and CTA examination were recommended, but she refused for personal reasons. Telephone follow-up at 6 months and 1 year indicated that there were no abnormalities in her hand function.

## Discussion

The incidence of and optimal treatment for arterial rupture, a rare and potentially lethal complication of NF1,^4^^,^^5^ have not yet been reported. The NF1 gene is a tumor suppressor gene localized to chromosome 17q11.2,^1^^,^^6^ which encodes the protein neurofibromin. Abnormality or deletion of this protein owing to gene mutation results in the occurrence of NF1.^6^^,^^7^ Common pathological changes of NF1 include characteristic cutaneous café-au-lait spots, lack of pigment in the iris, optic glioma, neurofibroma, and distinctive changes in the sphenoid wing or long bone.^2^^,^^8^

Vascular complications are rare in patients with NF1. The incidence of arterial complications in patients with NF1 ranges from 0.4% to 7.4%.^9^, ^10^, ^11^, ^12^ The types of lesions include arterial stenosis,^11^^,^^13^^,^^14^ arterial dissection,^15^, ^16^, ^17^ arteriovenous fistula,^18^, ^19^, ^20^ aneurysm, and, very rarely, arterial rupture, which carries a fatality rate second only to that of NF1-related malignancies.^6^^,^^21^ According to our literature review, only 64 cases of NF1-related arterial rupture have been reported between 1996 and 2024. By our analysis, the male-to-female ratio was approximately 1:1.5 (25:39); 85% of the cases occurred in young and middle-aged patients (30-60 years old), which is consistent with previous conclusion.^21^ Rupture sites are listed in Table I, the cervicothoracic arteries (including the cervical artery, extracranial vertebral artery, the subclavian artery, the intercostal artery and the mammary artery, etc) was most common affected, accounting for 47.1% of all cases, followed by the abdominal-pelvic arteries; rupture of the limb arteries was rare (10%). In most cases, there is only one rupture site, like the case 2; however, in rare cases, there are more rupture sites, like in case 1. Higa et al^22^ reported a NF1 patient who suffered arterial rupture of the renal artery, L4 lumbar artery, and deep circumflex iliac artery sequentially and underwent three different interventional operations within 24 days.

**Table I:** Rupture sites and cases number reported between 1996 and 2023

Rupture site	Number
Subclavian artery and its small branches	15
Carotid artery	4
Vertebral artery (extracranial segment)	7
Occipital artery	1
Internal thoracic artery	2
Intercostal artery	4
Internal mammary artery	1
Aorta/AA/AD	4
Aorta after EVAR	1
Pancreaticoduodenal artery	1
SMA	3
Colic aneurysm	1
Splenic artery	1
Renal artery	4
Lumbar artery	3
Iliac artery	2
Internal iliac artery	2
Superior rectal artery	3
Internal pudendal artery	1
Upper limb arteries	4
Lower limb arteries	3
Arteriovenous fistula	2
Pulmonary bulla	1

*AA,* Aortic aneurysm; *AD,* aortic dissection; *EVAR,* endovascular aorta repair; *SAM,* superior mesenteric artery.

It is believed that NF1 gene mutation induces dysplasia of the vascular smooth muscle and connective tissue, which causes the structure of vessels to be loose and brittle.^18^^,^^23^^,^^24^ Meanwhile, the elastic fibers reduction caused intima or media decrease,^25^ endothelial dysfunction caused arterial wall sclerosis,^26^ all together ultimately increasing the arterial fragility and the risk of spontaneous rupture.^7^^,^^26^ These pathological changes also explain why the NF1 patients' vessels tend to develop rupture-prone aneurysms.^27^

According to its pathological characteristics, we believe that NF1-artery rupture might result from mechanical stimulation, including muscle stretching. In both cases we report herein, the patients had a history of carrying loads or lifting heavy objects with the limb near the rupture sites before symptom onsetting. Similarly, Miyamoto et al^28^ reported that a NF1 patient turning backward to take a bag caused a tiny branch of the LSA rupture. Lee et al^6^ reported two NF1 patients with brachial aneurysm rupture, both of whom had a history of limb exertion or begin hit at the ruptured site.

Like other kinds of artery damage, emergency surgical hemostasis and arterial repair should be considered for NF1-related artery rupture to prevent shock and distal ischemia; optimal treatment for NF1-related artery rupture remains unclear.

Considering the fragile arteries of NF1 patients, incisional arterial repair may be extremely difficult. Just like our attempt in case 2, De Santis et al^29^ reported a failed brachial aneurysm rupture repair for a NF patient; the fragile artery could not tolerate suturing, they had to sutured the artery to the surrounding tissue to stop the bleeding. Hinsch et al^30^ reported that a NF1 patient's ruptured artery was too fragile and the surrounding tissues were unable to withstand tension; thus, significant bleeding was inevitable. A similar situation has been discussed in several articles: patients suffered uncontrollable bleeding^31^ or required a second surgery,^32^ even amputation.^33^ Most recently, Zhang et al^34^ reported disastrous bleeding when treating a NF1 patient with a necrotic subcutaneous hematoma after a intercostal artery rupture; multiple arteries had to be sutured.

Compared with traditional incisional arterial repair, endovascular repair is less invasive, safer, and allows intraoperative evaluation of the effect and distal limb's blood supplement.

Among the 64 NF1 patients who experienced arterial or aneurysm rupture between 1996 and 2023, 42 (65.6%) underwent endovascular procedures, most of whom had favorable prognoses. The specific techniques included stent implantation and coil or agent embolization, which should be chosen according to the patient condition. Of the 42 patients reported previously, 31 underwent the coil or harden agents (eg, polidocanol [Lauromacrogol] or gel) embolization, while the remaining patients underwent stent implantation with or without embolization, like Case1.

Certainly, the NF1 patients’ fragile arteries bring challenges to the endovascular procedure too. Just like the case 2, Doleman et al^35^ reported an extra stent implantation as bleeding when they trying to endovascularly passing through an extreme tortuous artery with a sharp angle. Lee et al^6^ reported that two NF1 patients who had to undergo amputation because the arteries reruptured after interventional embolization. Surgeons should consider the arterial fragility and handle the tortuous part of the artery with great care.

## Conclusions

The arteries of NF1 patients are fragile and weak owing to structural failure and are, therefore, prone to rupture. Endovascular procedures are advantageous in the treatment of NF1-related artery rupture and should be regarded as the first choice for intervention.^6^ It is important to perform endovascular procedures carefully, and surgeons must consider the potential risks of the fragile artery, and plan accordingly. Efforts to achieve incisional hemostasis must be prepared at all time. NF1 patients should be informed of the risks associated with their fragile arteries, and the importance of controlling blood pressure and avoiding aggravating activities. However, our conclusion was made based on previous case reports and our single-center experience. It needs to be supported by high-level evidence and long-term follow-up to standardize the treatment of the NF1-related arterial rupture.

LV and YG contributed equally to this article and share co-first authorship.

## Funding

None.
