# Supplementary material for: Spontaneous peripheral artery rupture in patients with neurofibromatosis type 1
Source: J Vasc Surg Cases Innov Tech. 2025 Jun 6;11(5):101873. doi: 10.1016/j.jvscit.2025.101873 (PMC12281292; doi:10.1016/j.jvscit.2025.101873)
Supplement: Supplementary Table [file mmc1.docx]

**Table 1. Type and treatment of artery rupture in patients with NF1 in 1996-2023**

| Rupture site | Age/Sex | Treatment | Remarks |
| --- | --- | --- | --- |
| SMA^#^ aneurysm | 44/M | Laparotomy artery ligation[1] | Prognosis not described |
| Vertebral artery | 56/F | Endovascular embolization[2] |  |
| Subclavian artery | 61/M |  | Patient died as sudden bleeding before operation [3] |
| Brachial artery | 30/F | Failed on autologous GSV^##^ transplantation, trans humeral amputation as re-bleeding[4] |  |
| Redial arterial aneurysm | 45/M | Incision hemostasis, hematoma clearance[5] |  |
| Internal carotid arterial aneurysm | 28/F | Endovascular coil embolism, stent implant[6] |  |
| Intercostal arterial aneurysm rupture caused hemothorax | 42/F | Endovascular coil embolism[7] |  |
| Aorta dissection with rupture | 34/M | Laparotomy aorta repairment[8] | Incision aorta suture, peritoneal encapsulation for hemostasis, second operation removing the fillers |
| Extracranial vertebral aneurysm | 52/F | Endovascular balloon occlusion, incisional ligation[9] | Patient died as cardiac arrest |
| Subclavian artery dissection with rupture | 48/M | Incision subclavian graft replacement[10] |  |
| Intercostal arterial aneurysm | 29/M | Endovascular embolism[11] |  |
| Internal thoracic artery | 48/F | Endovascular coil embolism [12] |  |
| Thyrocervical branch of subclavian artery | 76/F | Incision hemostasis[13] |  |
| Femoropopliteal aneurysm | 38/F | Incision hemostasis, autologous GSV^##^ transplantation[14] |  |
| Lumbar artery, renal artery, iliac artery and vein, inferior vena cava | 28/F | Laparotomy hemostasis[15] |  |
| infrarenal aorta | 49/F | Endovascular stent implant[16] |  |
| Intercostal artery | 48/M | Thoracotomy hemostasis[17] |  |
| Vertebral aneurysm,  Arteriovenous fistula | 60/F | Endovascular hemostasis[18] |  |
| Brachial aneurysm | 53/F | Incision hemostasis, autologous GSV^##^ transplantation[19] |  |
| Internal pudendal artery | 62/M | Endovascular embolism[20] |  |
| Profunda femoris aneurysm | 59/M | Incision artificial-vessel graft [21] |  |
| Superior mesenteric arterial aneurysm | 31/F | Endovascular covered stent implant[22] |  |
| Subclavian artery | 44/F | Endovascular stent implant[23] |  |
| Extracranial vertebral aneurysm and dissection | 36/F | Endovascular stent implant and coil embolism[24] |  |
| Branch of subclavian artery | 59/F | Incision arterial ligation[25] |  |
| Thyrocervical branch of subclavian artery | 47/F | Endovascular embolism[26] |  |
| Recurrent infrarenal aorta pseudoaneurysm after EVAR treatment | 49/M | Laparotomy stent take-out and artificial vessel repairment[27] |  |
| Recurrent rupture of intercostal artery aneurysm | 40/F | Endovascular embolism[28] |  |
| After-stent-implantation rupture of external iliac artery occlusion | 60/M | Endovascular stent implant[29] |  |
| Renal aneurysm | 41/F | Endovascular coil embolism[30] |  |
| Radial aneurysm | 48/F | Incisional decompression, hematoma evacuation[31] | Forearm aneurysm rupture result in osteo fascial compartment syndrome |
| Superior rectal aneurysm | 39/M | Endovascular embolism[32] |  |
| Costocervical trunk aneurysm (a branch of subclavian artery) | 43/M | Endovascular coil embolism[33] | The patient died of massive hemorrhage after surgery |
| Internal carotid artery | 66/F | Endovascular coil embolism[34] |  |
| Subclavian artery | 56/F | Endovascular Viabahn stent implant[35] |  |
| Internal iliac artery | 33/F | Laparotomy combining endovascular embolism[36] | The patient died of massive hemorrhage |
| Superior rectal artery aneurysm | 55/F | Endovascular embolism combining laparotomic hemostasis[37] |  |
| Internal carotid artery Neurofibromas | 42/M |  | Patient died before operation[38] |
| Extracranial vertebral artery rupture with arteriovenous fistula formation | 35/F | Endovascular coil embolism[39] |  |
| Internal thoracic aneurysm, hemothorax | 50/M | Endovascular embolism combining VATS^###^ [40] |  |
| Lumbar aneurysm | 52/M | Endovascular coil and drug embolism[41] |  |
| Internal mammary aneurysm | 55/F | Endovascular embolism[42] |  |
| Multiple occipital aneurysm | 53/F | Endovascular embolism[43] |  |
| Ascending aorta, arcus aorta | 42/F | Thoracotomy artificial arcus aorta replacement[44] | Pregnant patient |
| Accessory renal aneurysm | 44/F | Endovascular coil embolism[45] |  |
| Colic aneurysm rupture after EVAR | 67/F | Laparotomy left colon excision, transverse colostomy[46] |  |
| Extracranial vertebral aneurysm | 36/M | Endovascular coil embolism[47] |  |
| Vertebral artery rupture with hemothorax | 32/M |  | Patient died when angiography[48] [al] |
| Brachial aneurysm | 48/F | Incision arterial repairment[49] |  |
| Brachial aneurysm | 34/F | Conservative treatment at acute phase, delayed incision arterial repairment with autologous GSV transplant[50] |  |
| Splenic artery | 40/F | Endovascular embolism with stent implant[51] |  |
| Deep carotid artery | 49/M | Endovascular coil embolism[52] |  |
| Subclavian artery branch pseudoaneurysm | 33/M | Endovascular stent implant with liquid embolization[53] |  |
| PDA^####^ ＆SMA aneurysm | 55/F | Endovascular coil embolism[54] |  |
| Branch of brachial artery | 32/M | Endovascular embolism, shoulder amputation and repeat endovascular hemostasis after recurrent hemorrhage[55] | Patient died as massive hemorrhage |
| Branch of brachial artery | 33/M | Endovascular embolism, shoulder amputation as recurrent hemorrhage[55] |  |
| Internal iliac artery | 40/F | Endovascular coil embolism, ultrasound-guided aneurysm aspiration as aneurysm enlargement after endovascular procedure[56] |  |
| Renal artery-vena cava fistula, Lumbar artery | 59/M | Endovascular covered-stent implant and coil embolism[57] |  |
| Carotid aneurysm rupture after EVAR for aorta aneurysm | 78/F | Tracheostoma[58] |  |
| Superior rectal aneurysm | 52/F | Endovascular coil embolism[59] |  |
| Transverse cervical aneurysm rupture with airway obstruction | 52/M | Endovascular coil embolism[60] |  |
| Arterial malformation and rupture in lower limbs | 47/F | Endovascular embolism, above-knee leg amputation as unstoppable hemorrhage[61] |  |
| Rupture of bullae of lung result in hydrothorax | 18/M | Thoracic close drainage[62] |  |
| Infer renal aorta aneurysm | 49/F | EVAR^#####^[16] | Patient died after surgery |
|  |  |  |  |
|  |  |  |  |

# SMA: superior mesenteric artery;

## GSV: great saphenous vein;

### VATS: video-assisted thoracoscopic surgery

####PDA: superior pancreaticoduodenal artery

#####EVAR: endovascular aorta repairment

References

1. Huffman, J.L., V. Gahtan, V.D. Bowers, and J.L. Mills, Neurofibromatosis and arterial aneurysms. Am Surg, 1996. **62**(4): 311-4.

2. Horsley, M., T.K. Taylor, and W.A. Sorby, Traction-induced rupture of an extracranial vertebral artery aneurysm associated with neurofibromatosis. A case report. Spine (Phila Pa 1976), 1997. **22**(2): 225-7.

3. Miura, H., O. Taira, O. Uchida, J. Usuda, S. Hirai, and H. Kato, Spontaneous haemothorax associated with von Recklinghausen's disease: review of occurrence in Japan. Thorax, 1997. **52**(6): 577-8; discussion 75-6.

4. Tidwell, C. and P. Copas, Brachial artery rupture complicating a pregnancy with neurofibromatosis: a case report. Am J Obstet Gynecol, 1998. **179**(3 Pt 1): 832-4.

5. Singh, S., M. Riaz, A.D. Wilmshurst, and J.O. Small, Radial artery aneurysm in a case of neurofibromatosis. Br J Plast Surg, 1998. **51**(7): 564-5.

6. Smith, B.L., C.E. Munschauer, N. Diamond, and F. Rivera, Ruptured internal carotid aneurysm resulting from neurofibromatosis: treatment with intraluminal stent graft. J Vasc Surg, 2000. **32**(4): 824-8.

7. Kipfer, B., D. Lardinois, J. Triller, and T. Carrel, Embolization of a ruptured intercostal artery aneurysm in type I neurofibromatosis. Eur J Cardiothorac Surg, 2001. **19**(5): 721-3.

8. Chew, D.K., P.M. Muto, J.K. Gordon, A.J. Straceski, and M.C. Donaldson, Spontaneous aortic dissection and rupture in a patient with neurofibromatosis. J Vasc Surg, 2001. **34**(2): 364-6.

9. Miyazaki, T., F. Ohta, M. Daisu, and Y. Hoshii, Extracranial vertebral artery aneurysm ruptured into the thoracic cavity with neurofibromatosis type 1: case report. Neurosurgery, 2004. **54**(6): 1517-20; discussion 20-1.

10. Yoshida, K. and S. Tobe, Dissection and rupture of the left subclavian artery presenting as hemothorax in a patient with von Recklinghausen's disease. Jpn J Thorac Cardiovasc Surg, 2005. **53**(2): 117-9.

11. Chang, W.C., H.H. Hsu, H. Chang, and C.Y. Chen, Spontaneous hemothorax caused by a ruptured intercostal artery aneurysm in von Recklinghausen's neurofibromatosis. J Formos Med Assoc, 2005. **104**(4): 286-9.

12. Kim, S.J., C.W. Kim, S. Kim, T.H. Lee, K.I. Kim, T.Y. Moon, and S.W. Chung, Endovascular treatment of a ruptured internal thoracic artery pseudoaneurysm presenting as a massive hemothorax in a patient with type I neurofibromatosis. Cardiovasc Intervent Radiol, 2005. **28**(6): 818-21.

13. Ishizu, A., T. Ooka, T. Murakami, and T. Yoshiki, Rupture of the thyrocervical trunk branch from the subclavian artery in a patient with neurofibromatosis: a case report. Cardiovasc Pathol, 2006. **15**(3): 153-6.

14. Gutarra, F., J. Rodriguez Asensio, M. Miceli, and E. Mareso, Ruptured femoropopliteal artery aneurysms in von Recklinghausen neurofibromatosis. J Vasc Surg, 2007. **46**(4): 808-11.

15. Hinsch, N., S. Kriener, R.G. Ritter, and K. Holzer, Fatal haemorrhage due to extensive fragility of medium- and large-sized arteries and veins in a young patient with neurofibromatosis 1. Cardiovasc Pathol, 2008. **17**(2): 108-12.

16. Falcone, J.L., M.R. Go, D.T. Baril, G.J. Oakley, M.S. Makaroun, and R.A. Chaer, Vascular wall invasion in neurofibromatosis-induced aortic rupture. Vasc Endovascular Surg, 2010. **44**(1): 52-5.

17. Aizawa, K., C. Iwashita, T. Saito, and Y. Misawa, Spontaneous rupture of an intercostal artery in a patient with neurofibromatosis type 1. Interact Cardiovasc Thorac Surg, 2010. **10**(1): 128-30.

18. Higa, G., J.P. Pacanowski, Jr., D.T. Jeck, K.R. Goshima, and L.R. León, Jr., Vertebral artery aneurysms and cervical arteriovenous fistulae in patients with neurofibromatosis 1. Vascular, 2010. **18**(3): 166-77.

19. Emori, M., N. Naka, H. Takami, T.A. Tanaka, Y. Tomita, and N. Araki, Ruptured brachial artery aneurysm in a patient with type 1 neurofibromatosis. J Vasc Surg, 2010. **51**(4): 1010-3.

20. Zhang, C.W., Z.G. Yang, X.D. Xie, C.H. Wang, C. You, and W. Li, Transcatheter embolization of a ruptured internal pudendal artery pseudoaneurysm in a patient with neurofibromatosis type 1. J Korean Med Sci, 2010. **25**(4): 638-40.

21. Emrecan, B., G. Onem, and I. Susam, Ruptured profunda femoris aneurysm secondary to neurofibromatosis: vascular involvement in an unusual location. Tex Heart Inst J, 2010. **37**(3): 368-70.

22. Mendonça, C.T., J. Weingartner, C.A. de Carvalho, and D.S. Costa, Endovascular treatment of contained rupture of a superior mesenteric artery aneurysm resulting from neurofibromatosis type I. J Vasc Surg, 2010. **51**(2): 461-4.

23. Santin, B.J., G.E. Guy, E.C. Bourekas, and M.R. Go, Endovascular therapy for subclavian artery rupture in von Recklinghausen disease. Vasc Endovascular Surg, 2010. **44**(8): 714-7.

24. Morvan, T., F. de Broucker, and T. de Broucker, Subarachnoid hemorrhage in neurofibromatosis type 1: case report of extracranial cerebral aneurysm rupture into a meningocele. J Neuroradiol, 2011. **38**(2): 125-8.

25. Miyazaki, T., T. Tsuchiya, T. Tagawa, N. Yamasaki, and T. Nagayasu, Spontaneous hemothorax associated with von Recklinghausen's disease: report of a case. Ann Thorac Cardiovasc Surg, 2011. **17**(3): 301-3.

26. Hung, M.C., E. Yang, Y.C. Huang, and R.S. Chang, Spontaneous hemorrhage within the neck of a neurofibromatosis type 1 patient. J Emerg Med, 2012. **43**(3): 448-50.

27. Park, Y.J., K.M. Park, J. Oh, H.S. Park, J.S. Kim, and Y.W. Kim, Spontaneous aortic rupture in a patient with neurofibromatosis type 1. J Korean Surg Soc, 2012. **82**(4): 261-5.

28. Misao, T., T. Yoshikawa, M. Aoe, Y. Ueda, M. Yodoya, and J. Sakurai, Recurrent rupture of intercostal artery aneurysms in neurofibromatosis type 1. Gen Thorac Cardiovasc Surg, 2012. **60**(3): 179-82.

29. Doleman, B., S. Kaushal, A. Patel, J. Kirk, and J. Quarmby, Rupture of the Left External Iliac Artery and Right Groin Pseudoaneurysm Formation following Angioplasty in a Patient with Neurofibromatosis Type 1 and Undiagnosed Bilateral Phaeochromocytoma. Case Rep Radiol, 2013. **2013**: 526421.

30. Niwa, N., H. Yanaihara, M. Horinaga, Y. Nakahira, F. Hanashima, and H. Asakura, Spontaneous renal artery aneurysm rupture in a patient with neurofibromatosis type 1 without risk factors for renal artery aneurysm rupture. Vasc Endovascular Surg, 2013. **47**(7): 558-60.

31. De Santis, F., G. Negri, G. Martini, and G. Mazzoleni, Multiple aneurysms of the radial artery in a woman with neurofibromatosis type 1 presenting as aneurysm rupture. J Vasc Surg, 2013. **58**(5): 1394-7.

32. Makino, K., N. Kurita, M. Kanai, and M. Kirita, Spontaneous rupture of a dissecting aneurysm in the superior rectal artery of a patient with neurofibromatosis type 1: a case report. J Med Case Rep, 2013. **7**: 249.

33. Hoonjan, B., N. Thayur, and A. Abu-Own, Aneurysmal rupture of the costo-cervical trunk in a patient with neurofibromatosis type 1: A case report. Int J Surg Case Rep, 2014. **5**(2): 100-3.

34. Hamasaki, O., F. Ikawa, T. Hidaka, Y. Kurokawa, and U. Yonezawa, Extracranial internal carotid artery pseudoaneurysm associated with neurofibromatosis type 1 treated with endovascular stenting and coil embolization. Vasc Endovascular Surg, 2014. **48**(2): 176-9.

35. Mydin, M.I., A. Sharma, Z. Zia, M. Hawari, M. Jadoon, and A. Majewski, A novel approach in managing right-sided haemothorax in neurofibromatosis type 1. Asian Cardiovasc Thorac Ann, 2015. **23**(5): 573-5.

36. Moerbeek, P.R., J.M. van Buijtenen, B. van den Heuvel, and A.W. Hoksbergen, Fatal retroperitoneal bleeding caused by neurofibromatosis: a case report and review of the literature. Case Rep Med, 2015. **2015**: 965704.

37. Yow, K.H., J. Bennett, P. Baptiste, and P. Giordano, Successful combined management for ruptured superior rectal artery aneurysm in neurofibromatosis type 1. Ann Vasc Surg, 2015. **29**(6): 1317.e13-6.

38. Liang, Y., F. Tong, L. Zhang, W. Li, and Y. Zhou, Sudden death due to rupture of the right internal carotid artery in neurofibromatosis type 1: A case report. Leg Med (Tokyo), 2016. **21**: 33-7.

39. Uneda, A., K. Suzuki, S. Okubo, K. Hirashita, M. Yunoki, and K. Yoshino, Neurofibromatosis Type 1-Associated Extracranial Vertebral Artery Aneurysm Complicated by Vertebral Arteriovenous Fistula After Rupture: Case Report and Literature Review. World Neurosurg, 2016. **96**: 609.e13-09.e18.

40. Kwon, O.Y., G.J. Kim, T.H. Oh, Y.O. Lee, S.C. Lee, and J.Y. Cho, Staged Management of a Ruptured Internal Mammary Artery Aneurysm. Korean J Thorac Cardiovasc Surg, 2016. **49**(2): 130-3.

41. Ishigaki, T., R. Kawasaki, H. Matsuda, and N. Mukohara, Endovascular Treatment for a Ruptured Lumbar Artery Aneurysm in a Patient with von Recklinghausen Disease. EJVES Short Rep, 2018. **38**: 1-3.

42. Kim, D.W., I.S. Jeong, K.J. Na, B.S. Oh, B.H. Ahn, and S.Y. Song, Successful treatment of a ruptured left internal mammary artery aneurysm with a delayed diagnosis of type I neurofibromatosis. J Thorac Dis, 2017. **9**(9): E739-e42.

43. Bissacco, D., M. Domanin, S. Romagnoli, E. Martelli, V. Civelli, and L. Gabrielli, Spontaneous Rupture of Multiple Occipital Artery Aneurysms in a Patient With Neurofibromatosis Type 1. Vasc Endovascular Surg, 2018. **52**(1): 86-88.

44. Tateishi, A., M. Okada, M. Nakai, Y. Yokota, and Y. Miyamoto, Spontaneous ascending aortic rupture in a pregnant woman with neurofibromatosis type 1. Gen Thorac Cardiovasc Surg, 2019. **67**(11): 979-81.

45. Roberts, K., B. Fan, and R. Brightwell, Spontaneous Accessory Renal Artery Aneurysm Rupture in a Patient With Neurofibromatosis Type 1: A Case Report. Vasc Endovascular Surg, 2019. **53**(2): 150-53.

46. Moro, K., H. Kameyama, K. Abe, J. Tsuchida, Y. Tajima, H. Ichikawa, et al., Left colic artery aneurysm rupture after stent placement for abdominal aortic aneurysm associated with neurofibromatosis type 1. Surg Case Rep, 2019. **5**(1): 12.

47. Han, K.S., K.M. Lee, B.J. Kim, B.D. Kwun, S.K. Choi, and S.H. Lee, Life-Threatening Hemothorax Caused by Spontaneous Extracranial Vertebral Aneurysm Rupture in Neurofibromatosis Type 1. World Neurosurg, 2019. **130**: 157-59.

48. Bidad, R., C. Hall, and E. Blohm, Fatal Tension Hemothorax Combined With Exanguination: A Rare Complication of Neurofibromatosis. Clin Pract Cases Emerg Med, 2019. **3**(4): 364-68.

49. Degenaar, K.J., B. Barvelink, S. Nienhuis, and B.H. Elsman, Successful Surgical Reconstruction of a Ruptured Brachial Artery Aneurysm in a Patient With Type 1 Neurofibromatosis. EJVES Short Rep, 2019. **43**: 18-20.

50. Balanescu, C., R. Brar, N. Evans, J. Tsui, B. Lindsey, A. Papadopoulou, et al., Successful Repair of a Vasculopathic Aneurysmal Brachial Artery in a Patient with Type 1 Neurofibromatosis. Ann Vasc Surg, 2019. **61**: 467.e17-67.e22.

51. Morita, R., D. Abo, T. Soyama, Y. Yoshino, T. Yoshikawa, T. Kimura, et al. Spontaneous rupture of the pancreatic arcade artery caused by neurofibromatosis type 1 successfully treated using emergency transcatheter arterial embolization, partial intra-aortic balloon occlusion, and stent graft placement: a case report and review of the literature. CVIR Endovasc, 2020. **3**(1): 37.

52. Miyamoto, K., M. Nakamura, K. Suzuki, S. Katsuki, Y. Kaki, G. Inoue, et al., Diagnosis of neurofibromatosis type 1 after rupture of aneurysm and consequent fatal hemothorax. Am J Emerg Med, 2020. **38**(7): 1543.e3-43.e5.

53. Negreira, K.E., J.P. Lichtenberger, 3rd, B. Allais, A. Alhaddad, M. Bernetich, and V. Jain, Subclavian Artery Branch Pseudoaneurysm Rupture With Massive Hemothorax in a Patient With Neurofibromatosis Type 1. Chest, 2020. **157**(4): e103-e05.

54. Fukushima, N., H. Aoki, S. Takenaga, K. Morikawa, M. Ogawa, and K. Yanaga, Ruptured visceral artery aneurysms in a patient of neurofibromatosis type 1 (NF-1) successfully treated by endovascular treatment. Surg Case Rep, 2020. **6**(1): 18.

55. Lee, J. and Y. Kim, Life-threatening brachial artery hemorrhage and a lethal outcome in patients with neurofibromatosis type 1: two case reports and a review of the literature. J Int Med Res, 2021. **49**(6): 3000605211025344.

56. Kamada, K., A. Koya, A. Tochikubo-Suzuki, S. Kikuchi, D. Uchida, and N. Azuma, Successful endovascular therapy involving direct puncture for spontaneous internal iliac artery aneurysm rupture. J Vasc Surg Cases Innov Tech, 2022. **8**(1): 125-28.

57. Higa, S., T. Nagano, J. Ito, A. Uejo, M. Nakaema, Y. Kise, et al. Three Arterial Ruptures in a Patient with Neurofibromatosis Type 1. Ann Vasc Dis, 2021. **14**(2): 168-72.

58. Nakai, S., T. Uchida, Y. Kuroda, A. Yamashita, E. Ohba, M. Mizumoto, et al., Endovascular Repair for Abdominal Aortic Aneurysm Rupture With Neurofibromatosis Type 1. Ann Vasc Surg, 2022. **79**: 439.e1-39.e4.

59. Nemoto, H., K. Mori, Y. Takei, S. Kikuchi, S. Hoshiai, Y. Yamamoto, et al. Treatment of ruptured rectal artery aneurysm in a patient with neurofibromatosis. CVIR Endovasc, 2022. **5**(1): 37.

60. Okazaki, Y., T. Ichiba, and N. Fujisaki, Potential Fatal Complication of Neurofibromatosis Type 1: Acute Upper Airway Obstruction Due to Ruptured Transverse Cervical Artery Aneurysm. Cureus, 2022. **14**(12): e32910.

61. Shen, L.P., G. Jin, R.T. Zhu, and H.T. Jiang, Hemorrhagic shock due to ruptured lower limb vascular malformation in a neurofibromatosis type 1 patient: A case report. World J Clin Cases, 2022. **10**(31): 11597-606.

62. Guerra, M., I. Farinha, and D. Marado, Spontaneous Primary Pneumothorax as A Complication of Neurofibromatosis Type 1. Eur J Case Rep Intern Med, 2023. **10**(11): 004046.
